# Connexin Gap Junctions and Hemichannels in Modulating Lens Redox Homeostasis and Oxidative Stress in Cataractogenesis

**DOI:** 10.3390/antiox10091374

**Published:** 2021-08-28

**Authors:** Yumeng Quan, Yu Du, Yuxin Tong, Sumin Gu, Jean X. Jiang

**Affiliations:** Department of Biochemistry and Structural Biology, University of Texas Health Science Center, San Antonio, TX 78229, USA; quany@uthscsa.edu (Y.Q.); duy2@uthscsa.edu (Y.D.); tongy1@uthscsa.edu (Y.T.); gus@uthscsa.edu (S.G.)

**Keywords:** oxidative stress, connexin, gap junction, hemichannel, lens, redox homeostasis, cataractogenesis

## Abstract

The lens is continuously exposed to oxidative stress insults, such as ultraviolet radiation and other oxidative factors, during the aging process. The lens possesses powerful oxidative stress defense systems to maintain its redox homeostasis, one of which employs connexin channels. Connexins are a family of proteins that form: (1) Hemichannels that mediate the communication between the intracellular and extracellular environments, and (2) gap junction channels that mediate cell-cell communication between adjacent cells. The avascular lens transports nutrition and metabolites through an extensive network of connexin channels, which allows the passage of small molecules, including antioxidants and oxidized wastes. Oxidative stress-induced post-translational modifications of connexins, in turn, regulates gap junction and hemichannel permeability. Recent evidence suggests that dysfunction of connexins gap junction channels and hemichannels may induce cataract formation through impaired redox homeostasis. Here, we review the recent advances in the knowledge of connexin channels in lens redox homeostasis and their response to cataract-related oxidative stress by discussing two major aspects: (1) The role of lens connexins and channels in oxidative stress and cataractogenesis, and (2) the impact and underlying mechanism of oxidative stress in regulating connexin channels.

## 1. Introduction

The lens is an avascular and transparent organ in the anterior segment of the eye. Its primary function is to transmit and focus light onto the retina. The lens transmits various wavelengths of light and filters out almost all ultraviolet (UV) light. Lenses are constantly exposed to UV radiation, which generates reactive oxygen species (ROS) and causes photochemical damages to biological molecules [1,2]. ROS, including superoxide anion (O_2_^•−^), hydroxyl (HO^•^), singlet oxygen (^1^O_2_), and hydrogen peroxide (H_2_O_2_), cause oxidative modifications of proteins, resulting in protein function loss and aggregation, and ultimately cataract formation [2]. To maintain redox homeostasis under continuous oxidative insults, the lens develops an oxidative stress defense mechanism with powerful capacities to modulate redox metabolism. This complex antioxidative mechanism includes simple ROS scavengers and more advanced enzyme protective systems [3,4]. Moreover, an internal lens microcirculation system composed of connexin channels, ion channels, and ion pumps mediates the transport of nutrition and metabolites. It also plays a critical role in protecting the lens against oxidative damage [5]. Under physiological or pathological conditions, connexin channels are regulated and modified in response to several oxidative insults and can, in turn, regulate the redox state [6]. In this review, we will discuss the important roles of connexin channels in the lens under oxidative stress and cataractogenesis, focusing on regulating connexin-forming gap junction intercellular communication (GJIC) and hemichannels (HCs) under physiological or pathological conditions. We aim to provide a summary of recent research advances in our understanding of mechanical roles of connexin channels in lens redox homeostasis and oxidative stress-induced lens disorders.

## 2. Connexins and Intercellular Communication in the Lens

### 2.1. Expression of Connexin Isoforms in the Lens

Connexins are a family of membrane proteins containing four transmembrane domains, two extracellular loop domains, and one cytoplasmic loop domain. The N- and C-termini are located in the cytoplasm [7]. Six connexin molecules are intracellularly oligomerized and assembled into a hemichannel (also called connexons) and then transported to the plasma membrane. Hemichannels can consist of either the same connexin isoforms or a combination of different connexin isoforms, referred to as homomeric or heteromeric hemichannels, respectively [8]. Two hemichannels from two adjacent cells dock with each other in the extracellular space to form a complete gap junction channel [9]. Two adjacent cells contribute different types of hemichannels, which can pair either with a single type or different homomeric or heteromeric hemichannels (connexons), to form homotypic channels and heterotypic channels, respectively. However, not all connexins can form functional heteromeric or heterotypic channels; for instance, Cx43 can form heterotypic channels with Cx50, but not with Cx46 [10]. The formation of these complex structures depends on the compatibility of connexins composing the channels. In turn, the connexin composition in the channels influences the conductance, permeability, and selectivity of the channels [11,12]. 

The ocular lens is comprised of two cell types—epithelial cells that form a single layer along the lens anterior, and fiber cells that make up the bulk of the lens organ. Three connexin isoforms have been identified in the lens with different spatial expression patterns. Cx43 is expressed in the lens epithelium, but not fiber cells, and its expression is downregulated as the epithelium differentiates into fiber cells in the equatorial lens region. Cx46 is absent in epithelial cells, but becomes highly expressed during fiber cell differentiation. In comparison, Cx50 is initially synthesized in the epithelium and remains at high levels in differentiating fiber and mature fiber cells [13,14,15]. Interestingly, a recent study by Gong et al. [16] reported that human and mouse lenses have different distribution patterns of connexin proteins. For example, in contrast to the mouse lens, where Cx46 is primarily expressed in the fiber cells [17,18], both Cx46 and Cx50 in the human lens are expressed in the epithelial cells of transparent and cataractous lenses. Gong et al. [16] further reported that the ratio between Cx43 and Cx46/Cx50 in humans is 9:1, which is higher than in mice (5:1), indicating a different expression pattern of connexin isoforms. Although transcripts for a fourth connexin, Cx23, have been detected in the zebrafish embryo and mouse lens, there is no report for its expression in the human lens [19,20]. Therefore, Cx23 will not be further discussed in the present review. It should provide a concise and precise description of the experimental results, their interpretation, and the experimental conclusions that can be drawn.

### 2.2. Connexin Channels and Lens Microcirculation

Connexin channels allow the passage of molecules with size no more than 1 kDa [21], and provide a pathway for the intercellular and intracellular exchange of ions, small metabolites, and second messengers, such as Na^+^, K^+^, Ca^2+^, cAMP, cGMP, inositol trisphosphate (IP_3_), ADP, ATP, prostaglandin E_2_ (PGE_2_), glucose, and glutathione [12,22,23,24]. Although under certain physiological conditions, few unopposed HCs are open, studies show that HCs can open and act as channels, independently from gap junctions. HCs can open at the plasma membrane under physiological and pathophysiological conditions in several cell types, including lens cells and bone osteocytes [21,25].

As an optical element in the light pathway, the lens maintains transparency and has evolved a unique structure to minimize light scattering through organelle degradation and loss [26]. Interestingly, the mature fiber lacking organelles survive throughout their entire life without *de novo* synthesis of proteins. Hence, transporting nutrition and metabolites between mature fiber cells is crucial for their survival [1]. An internal transport model has been proposed by Mathias et al. [27] that the lens operates a microcirculatory system that delivers water, ions, and solute for lens cells. In this model, the current enters the lens and the extracellular spaces between cells at both the anterior and posterior poles, and exits at the lens equator [1,28]. Fiber cell gap junctions are one of the major components of the lens microcirculation model [5]. The equatorial differentiating fiber cells are postulated to regulate the angular variation in conductance through an fibroblast growth factor (FGF)-mediated increase of the number of open gap junction channels [29]. This model is consistent with the distribution of ion channels and pumps in the lens [28]. This system is critical for maintaining the optical properties and enhancing the transparency of the lens. Studies show that gap junction conductance plays an important role in the microcirculation of the lens and that GJIC modulates lens microcirculation through lens intracellular hydrostatic pressure. [30,31,32]. Ebihara et al. [33] report that Cx46 HCs may play an important role in the lens internal circulation system by allowing the entry of sodium from the extracellular space into lens fiber cells. Connexin HCs on the surface of lens fiber cells are subjected to fluid flow shear stress (FFSS) applied by mechanical loading, which is caused by lens accommodation and constitutive microcirculation [5]. Our recent study provides direct evidence that glucose and antioxidants are efficiently transported through lens microcirculation mediated by Cx50 HC opening induced by FFSS. It indicates HCs as a potential, major delivery portal for nutrients and antioxidants in lens fiber cells [34]. In addition, recent studies show that mutations in Cx50 and Cx46 cause cataracts by compromising lens circulation through calcium accumulation and precipitation [35,36,37].

## 3. Oxidative Stress and Cataractogenesis

### 3.1. Oxidative Damage in Cataract Formation

Oxidative stress plays an important role in various aging-related pathogenetic processes and diseases. For example, oxidative free radicals induce cumulative and irreversible cellular malfunction and even cell death [38]. Oxidation of proteins, lipids, and DNA has been demonstrated in cataractous lenses [39,40,41,42,43]. In the most advanced cataracts, over 90% of protein sulfhydryl (-SH) groups are lost, and almost half of the methionine residues in the nuclear protein become oxidized to methionine sulfoxide [44]. Oxidation-related modifications, including protein insolubilization/loss of soluble protein, aggregation, and non-disulfide crosslinking, are likely caused by a homeostatic imbalance of the redox state in the lens. Various lines of evidence suggest the loss of glutathione (GSH) and the significant increase of oxidized GSH (also known as glutathione disulfide, GSSG) levels in age-related cataract development [45,46].

Besides aging, many other risk factors play major roles in cataractogenesis, such as ultraviolet light, hyperglycemia, tobacco, and selenite [44,47,48,49,50,51,52,53,54]. The lens is capable of transmitting light of various wavelengths and filtering out almost all UV light. In response to strong, continuous UV radiation, this filtering function can be compromised, leading to lens protein damage and the development of cataracts [55,56,57]. Ultraviolet B (UVB) is responsible for photochemical reactions that damage the lens through the generation of ROS [57,58]. The free UV filters (tryptophan derivatives) decrease with aging, which causes the lens to be progressively more susceptible to UV damage and oxidation [48]. Many studies report increased free radicals, impaired antioxidant capacity, and increased susceptibility to oxidative stress in diabetic lenses, suggesting that high glucose induces cataracts through increased oxidative stress [59,60].

### 3.2. Redox System in the Lens

ROS can be generated endogenously in different cellular compartments during normal metabolism through the enzymatic activities of lipoxygenases, NADPH oxidase, cytochrome P450, and mitochondrial electron transport, or through the effects of exogenous factors [61]. One of the most important and fundamental sources of endogenous ROS is the formation of O_2_^•−^ via the electron transport chain in the mitochondria [62,63]. The production of O_2_^•−^ can increase multiple damaging ROS species and promote redox imbalance in the lens. Moreover, because of the small size, some of the ROS generated outside the lens can pass through the plasma membrane of lens cells and lead to increased oxidative stress [64].

To maintain redox homeostasis and transparency, the lens develops a powerful antioxidant defense system containing several non-enzymatic and enzymatic mechanisms that protect the lens and repair damaged cell components. The lens possesses one of the highest tissue levels of reduced GSH (~4–6 mM), which represents the primary reducing system in the lens [65]. Other well-studied and important ROS scavengers in the lens are ascorbate (vitamin C) and vitamin E, crystallin protein chaperones, and free UV filters [45,66,67,68,69]. ROS are also degraded through the activities of enzymatic antioxidants, including superoxide dismutase (SOD), glutathione peroxidase (GPX), peroxiredoxins, microperoxidases, and catalase (CAT) [70,71,72]. GSH is converted to its oxidized form, GSSG, while GSSG can be regenerated back to its reduced form by glutathione reductase (GSR). Under normal cellular conditions in the lens epithelium, GSH is almost entirely found in its reduced state with barely detectable levels of GSSG [73,74]. A high level of reduced GSH is also found in healthy lens fibers cells, and likely is imported through connexin channels considering their lack of synthetic enzymes, due to degeneration of organelles [31,45,75,76,77,78].

As aforementioned, several protein modifications occur due to oxidative stress and lead to the formation of the high molecular weight insoluble protein aggregates [79,80,81]. After oxidative damage, lens protein repair requires the participation of the GSH-dependent thioltransferase, the NADPH-dependent thioredoxin/thioredoxin reductase system, and the methionine sulfoxide reductases [38]. A previous study has reported that deficiency of lens repair systems results in loss of mitochondrial membrane potential and increased ROS levels in lens cells [82].

In summary, cataractogenesis is a complex and multifactorial pathological process, and oxidative stress plays a critical role in the process. Antioxidant defense systems and protein repair mechanisms are developed to maintain redox homeostasis and transparency of the lens.

## 4. Oxidative Stress and Connexin Channels in Cataractogenesis

### Connexin Channels in Cataract Formation

Connexin-formed gap junctions and hemichannels in the lens mediate the small molecule fluxes according to their chemical concentration gradients [5,83]. Gong et al. [16] recently found that Cx43 is significantly upregulated by almost 50% in cataractous lenses, while both Cx46 and Cx50 are downregulated. Moreover, Cx50 is downregulated more than 90%, much greater than Cx46, in the fiber cells of ≥50-year-old human lenses. The altered expression patterns during aging suggest that age-dependent loss of Cx46 and Cx50 could contribute to senile cataractogenesis. It is also reported that there was no significant difference in the amounts of either Cx46 or Cx50 during selenite-induced cataract formation, while a decrease of Cx46 phosphorylation and an increase of cleaved Cx50 were observed [84]. 

An earlier study reported that there are no detectable abnormalities of lens transparency and lens fiber differentiation in Cx43-knockout mice [85]. In neonatal Cx43 (−/−) lenses, fiber cells exhibit largely separated apical surfaces of epithelial cells, grossly dilated extracellular spaces, and intracellular vacuoles between fiber cells [85,86]. Although Cx43 deletion does not influence prenatal lens development, the changes in Cx43 knockout mice are associated with early-stage cataract formation and indicate its unique, diverse function in regulating postnatal growth and homeostasis [85,86]. Since Cx43 knockout mice die shortly after birth, due to blockage of the ventricular outflow from the heart [87], it is challenging to observe postnatal cataract development and lens homeostasis. Most identified Cx43 single-point mutations in humans are correlated with several abnormalities, including oculodentodigital dysplasia, visceroatrial heterotaxia, hypoplastic left heart syndrome, and atrial fibrillation [88], but only one is associated with a lens disorder. These studies suggest that Cx43 dysfunction may not cause abnormalities of embryonic lens development. The Cx43 Y17S mutation has previously been associated with cataracts in oculodentodigital syndrome [89]. Furthermore, a study by Lai et al. [90] showed that the Y17S mutation in Cx43 reduces the activity of gap junctions and HCs in C6 cells compared to wild type Cx43. These observations suggested that Cx43 Y17S mutation-induced cataract formation is due to partial loss-of-function of connexin channels. Interestingly, our recent study revealed that the haploinsufficiency of Cx43 elevated oxidative stress and promoted susceptibility to cataracts in the mouse lens (unpublished data). However, the mechanical role of lens epithelial Cx43 in cataractogenesis remains largely elusive.

There have been ample studies with regards to the type of cataracts induced by genetic alterations of connexins, including nuclear, nuclear pulverulent, zonular pulverulent, finely granular embryonal, coppock-like and posterior polar [3,91,92,93,94]. Our recent study also provides evidence that connexin channels likely play a role in oxidative stress-related protein aggregation [95], which offers a potential mechanism in protecting lens proteins after oxidative damage. However, there are limited studies that investigate the role of connexins in the lens in response to oxidative stress in vivo. One study reported that deletion of protein kinase C gamma (PKCγ), an oxidative stress sensor [96,97], abolishes Cx50 phosphorylation on serine and threonine residues in the lens, eliminates the effects of uncoupling of Cx50 gap junctions upon H_2_O_2_ treatment, and decreases the frequency of gap junctions in lens cortical fiber cells, which, in turn, increase the susceptibility to oxidative damage of the lens [98]. Recently, two mouse connexin gene knockin models were developed employing two connexin mutants—Cx46fs380 and Cx50D47A. In these two models, connexin expression is decreased [99,100], and GJIC is attenuated both in differentiating and mature fibers. By detecting gap junction (GJ) coupling conductance, coupling conductance per area of radial contact between fiber cells was calculated from the reciprocal of resistivity multiplied by fiber cell width [36,101]. Since the effective intracellular resistivities are dominated by the resistance of GJs, they are inversely proportional to the number of opening GJs per area of radial cell-cell contact. GSH is reported to be transported through GJIC, and as a result of functional GJIC and other antioxidants mechanisms, oxidative damage is not an early critical event in cataractogenesis [102]. In this study [102], the GSH level unexpectedly does not decrease in Cx50D47A (2.5 months) and Cx46fs380 (4.5 months) mice lenses, while the level of glutathione synthetase is increased. These results indicate an unnecessary correlation between GSH level and the early stages of cataractogenesis. However, the data contradicts the previous concept of the association between decreased GSH levels and cataract formation. These studies further suggest the involvement of the p62-dependant antioxidant response [103], and the possible compensatory effects in response to the impaired differentiation in Cx50D47A mice [100], where the elevated GSH synthetase activity and GSH level are required to support the remaining organelles.

## 5. Connexin Channels in Response to Oxidative Stress

### 5.1. The Role of Connexin Channels in Response to Oxidative Stress

Previous studies have shown the biphasic, protective, and detrimental roles of Cx43 channels in modulating oxidative stress-induced cellular damage in several different tissues [78,104,105,106,107,108,109,110,111,112,113,114,115]. Whether connexin channels exert a protective or destructive effect in response to oxidative stress is dependent on the insults and cell and tissue types. Our previous work has demonstrated a protective mechanism mediated by Cx43 channels against oxidative stress in bone osteocytes [108]. In retinal pigment epithelial cells, Cx43 GJIC protects cells against the chemical oxidant tert-butyl hydroperoxide (t-BOOH)-induced cells death [107]. Selective inhibition of Cx43 HCs by Gap19, a selective Cx43 inhibitor of HCs, protects human umbilical vein endothelial cells from lipopolysaccharide-induced apoptosis [113]. Cigarette smoke extract and H_2_O_2_ lead to membrane depolarization and opening of HCs; this, in turn, likely predisposes epithelial cells to injury and apoptosis [112]. Chi et al. [115] prove that efflux of GSH via Cx43 HCs contributes to the Ca^2+^ depletion-elicited disassembly of cell junctions. In cardiomyocytes, the translocation of Cx43 to the mitochondrial inner membrane exerts cardioprotection ischemic and hypoxic postconditioning [114,116]. Recent studies show that Cx43 HCs in cardiomyocyte mitochondria interact with ATP-sensitive potassium channels (mKATP) and protect cardiomyocytes against hypoxia/ischemia stress [117,118]. In contrast to the cardioprotection, mitochondrial Cx43 HCs facilitate mitochondrial Ca^2+^ entry and may trigger permeability transition and cell injury/death by using connexin-targeting peptides interacting with extracellular (Gap26) and intracellular (Gap19, RRNYRRNY) Cx43 domains [119,120], indicating a detrimental role of Cx43 HCs in cardiomyocytes. In astrocytes, Cx43 deficiency or Cx43 channel inhibition resulted in increased ROS-induced astrocytic death, supporting a protective effect of Cx43 channels [105]. The viability of astrocytes is reduced in the hypoxia/reoxygenation process through increased permeability of Cx43 HCs and release of ATP and glutamate [121,122].

In response to environmental radiation and oxidants, ROS accumulates excessively in lens cells, including epithelial cells and the bulky fiber cells, as well as the surrounding fluids, such as the aqueous humor [123]. Connexins are subject to oxidative stress-induced post-translational modification, which can alter the conductance and activities of the lens connexin channels [96,124]. The previous study has shown that H_2_O_2_ leads to a dose- and time-dependent decrease of Cx46 in differentiated chick lens cultures [3]. A recent study demonstrated that glucose oxidase-induced oxidative stress causes significant upregulation of Cx43, and downregulation of Cx46 and Cx50 [16].

Our previous work has demonstrated a protective role of lens connexin channels against oxidative stress [78,125]. The Cx46 G143R missense mutation, associated with congenital Coppock cataracts, decreased Cx46 gap junctional coupling, and increased HC activity. Moreover, this mutation decreased the resistance of the cells to oxidative stress, primarily due to the increased HC function [125]. Recently, the study by Retamal et al. [126] further showed that Cx46 in the lens was carbonylated by 4-Hydroxynonenal (4-HNE) in a selenite-induced cataract animal model, suggesting that Cx46 is post-translationally modified by a lipid peroxide and that this modification reduces Cx46 HC activity. In lens fiber cells, Cx50 HCs open in response to H_2_O_2_ stimulation, which supports the cellular protective role of HCs against oxidative damage [78]. In this study, the dominant-negative mutants in Cx50, Cx50P88S (inhibiting both gap junctions and HCs), and Cx50H156N (only inhibiting HCs), block the protective role of Cx50 in response to H_2_O_2_-induced apoptosis, while the Cx50 E48K (only inhibiting gap junctions) does not have such effect. By using a Cx43E2 antibody, a specific Cx43 HC inhibitor [127], our group recently revealed that Cx43 HCs protect lens epithelial cells against oxidative stress (unpublished data). In contrast to the cell-protective effect, it has been reported that Cx43 HCs in lens epithelial cells play a detrimental role in linoleic acid-induced cell death [110]. This effect of HCs was validated by TATGap19, a specific Cx43 hemichannel inhibitor, but this study did not rule out the involvement of GJIC.

To date, most of the studies support the protective role of connexin channels against oxidative stress in the lens. The alteration in the expression of connexin isoforms in the lens during aging indicates that Cx43 may play a critical role in response to compromised redox homeostasis. On the other hand, connexin HCs opening in other cells/tissues has been shown to mediate the loss of GSH [115], implying a possible adverse effect on the lens under certain conditions.

### 5.2. Cellular Communication through Connexin Channels under Oxidative Stress

As an avascular organ, most of the lens’ metabolic needs rely on the aqueous humor and anterior epithelial cells. Lens fiber cells have limited antioxidant capabilities and require the import of antioxidant compounds like GSH, which are produced by lens epithelial cells. Connexin channels permit the transfer of ions and molecules less than 1 kDa [128], therefore permit delivery of reductant molecules to maintain a reduced environment in the lens fibers. The transport of such antioxidants and their oxidation molecules between the epithelial cells at the anterior surface of the lens and the bulk of lens fibers is through a microcirculation system, a network of membrane channels that includes connexin channels [5].

Several studies have shown that connexin channels are permeable to ROS and GSH [76,78,112,129,130,131]. Thus, it is expected that connexin channels play a pivotal role in maintaining the redox homeostasis of the lens. Furthermore, the oxidized form of GSH, GSSG, is believed to diffuse back to the lens periphery, where it can be reduced to regenerate GSH. The permeability of Cx43, Cx46, and Cx50 in the lens was investigated in a study by Slavi et al. [76]. They found that deletion of Cx46 led to a marked and selective decrease in GSH levels in the lens core, indicating that Cx46, but not Cx50, is necessary for the transport of GSH to the lens nucleus. However, GSSG permeation through fiber cell gap junction channels was undetectable in these studies. Contrary to GSH transport under normal conditions, we found that under oxidative stress conditions, Cx50 HCs protect lens fiber cells against H_2_O_2_-induced cell death by uptaking exogenous GSH [78]. Our recent study also indicates that Cx43 HCs in lens epithelium could uptake exogenous GSH and release GSSG in response to oxidative stress. Interestingly, we also found that Cx43 HCs protect epithelium through mediating H_2_O_2_ influx against H_2_O_2_-induced oxidative stress (unpublished data). These findings imply that the entry of ROS not only facilitates the acceleration of redox imbalance [112], but also triggers cellular antioxidant activities under certain oxidative conditions. Connexin channels are likely to maintain the redox hemostasis by redistributing oxidants/antioxidants with neighboring cells via GJIC or the extracellular microenvironment via HCs and preventing intracellular ROS accumulation.

### 5.3. Regulation of Lens Connexin by Oxidative Stress

Communication via connexin channels is dynamically regulated at multiple levels, which is achieved by changes in the unitary conductance of single-channels or probability of channel opening, as well as the presence of channels on the plasma membrane affected by the rates of synthesis and assembly, post-translational modification and/or protein degradation [21]. It was thought that HCs remained closed until their docking with another HC to prevent undesired leakage. However, increasing evidence supports that unopposed HCs can open under physiological or pathological conditions [132]. This open probability allows connexin HCs to participate in several cellular processes. The modification and regulation of connexin channel gating mechanisms under physiological or pathological conditions have been well-reviewed [6,133,134,135,136,137]. Here, we will focus on the role of redox signaling as an intermediary of connexin channels in regulating the lens under oxidative stress. In recent years, growing evidence supports that the responses of connexin GJICs and HCs can be observed with various methods of oxidative stress induction, such as cigarette smoke extract, cadmium, nitric oxide, metabolic inhibition, and H_2_O_2_ [78,104,112,138,139,140,141,142,143,144]. We recently studied the effect of two major oxidative stress sources, H_2_O_2_ and UVB radiation, on connexin HCs in the lens, and found that connexin HCs open in response to either H_2_O_2_ or UVB radiation (unpublished data). All these observations suggest that connexin channels are activated in response to oxidative stress, but the question is how oxidative stress opens HCs. The likely oxidative stress-induced regulatory factors include protein kinases/phosphatase, pH, intracellular Ca^2+^, and changes of membrane potential.

The study by Contreras et al. [140] suggested that connexin channels can be modulated by two free radical scavengers, melatonin and trolox. Moreover, Retamal et al. [138] reported that Cx43 HC opening can be blocked by dithiothreitol (DTT), a membrane-permeable reductant, but this blockage was not observed with membrane-impermeable GSH. The results indicate that HC inhibition by reducing agents likely acts on the oxidized intracellular cysteine (Cys) residues. In addition, another study [104] detected the activities of GJIC and HCs in astrocytes under a proinflammatory cytokine and oxidative stress environment. They showed that the increase of HC activity could be inhibited by L-name (an inhibitor of the nitric oxide synthase) and DTT; however, the decrease in GJIC was unaffected by either of the reagents. The modification of HCs by redox potential was also confirmed in HeLa cells by metabolic inhibition [141] and in smooth muscle cells upon stimulation by constrictor phenylephrine [142]. Cys271 has been suggested as an oxidation site of Cx43 [142], and recently validated as an S-nitrosylation site activated directly by nitric oxide [144]. We further found that Cx43 HCs activation induced by UVB radiation is regulated by the intracellular redox state through a different cysteine residue, Cys260 (unpublished data). Different Cys groups are differentially oxidized and induce different modifications to channel properties [145]. Therefore, further research is required to understand the role of specific Cys groups at the connexin C-terminus and their effect on connexin channels upon oxidative insults, which will ultimately aid in our understanding of the role of redox in pathological and physiological processes.

It is known that under pathological conditions, connexins can be modified by increased ROS, and the protein phosphorylation induced by oxidative stress is considered the most common form of post-translational modification. Connexin molecules have multiple consensus phosphorylation sites, and the phosphorylation of connexins mostly occurs at the C-terminal region. The direct phosphorylation of connexins can regulate GJCs and HCs properties, as well as connexin trafficking, GJ assembly, and stability [146,147,148]. Generally, connexins can be directly phosphorylated by serine/threonine kinases or tyrosine kinases, including protein kinase C (PKC), MAP kinase (MAPK), cAMP-dependent protein kinase A (PKA), casein kinase (CK), p34cdc2, protein kinase G (PKG), Ca^2+^/calmodulin-dependent kinase II (CaMKII), and the tyrosine kinase Src. Reported phosphorylation sites of connexins, responsible kinases, and the effect on GJIC and HCs have recently been described and summarized by Pogoda et al. [6]. Here, we primarily focus on oxidative stress-correlated connexin phosphorylation in the lens. 

It has been reported that PKC-dependent connexin phosphorylation decreases GJ conductance or dye coupling [149], and inhibition of PKC increases Cx43 HC activity [150]. The correlation between activities of GJIC and PKC-dependent Ser368 phosphorylation of Cx43 has been established in various cell types [6]. In ovine lens epithelial cells, TPA induced a gradual disappearance of Cx43 gap junction plaques and a transient decrease in Cx43 levels [151]. In rabbit lens epithelial cells, PKCγ phosphorylates Cx43, and this phosphorylation causes disassembly and loss of gap junction plaques from the cell surface [152]. Contrary to PKCγ, overexpression of PKCα leads to an increase in cell surface expression of Cx43. Furthermore, it has been reported by Lin and Takemoto [96] that H_2_O_2_-induced oxidation of the C1 domain activates PKCγ, and inhibits gap junctions. Similar increases in the phosphorylation of Cx46 and Cx50 were observed in rat lenses treated with H_2_O_2_ [124].

In addition to the most studied PKC, several studies have reported PKA kinase activity in the lens, including C43 phosphorylation at its C-terminal region [146,149]. There are also multiple in vivo phosphorylation sites of bovine Cx50 and Cx46 identified by mass spectrometry [153]. In earlier studies, Walsh and Patterson [154] reported that the topical administration of PKA activators, forskolin or 8-Br-cAMP, increased the equatorial current of the lens, although the substrates and the process remained unclear. Berthoud et al. [155] demonstrated constitutive phosphorylation of the Cx46 C-terminus in primary chicken lentoids. Both PKC and PKA efficiently phosphorylated GST-Cx56CT at S493 in vitro. A previous study by our laboratory [156] conducted mass spectrometry analysis and found that Ser395, located at the PKA consensus site at the C-terminus of chicken Cx50 was phosphorylated in the lens. Furthermore, analysis of Cx50 phosphorylation by two-dimensional thin-layer chromatography with tryptic phosphopeptide profiles suggested that Ser395 was directly phosphorylated by PKA in vivo. Moreover, PKA activation enhanced both Cx50 gap junction and HC function. Our study further confirmed that the enhanced GJIC did not involve increased Cx50 trafficking to the cell surface, Cx50 expression, or gap junction plaque formation, but gap junctions did appear to be stabilized in a more conductive configuration by single-channel recordings [156]. PKA enhanced transitions between the closed and open state (~200 ps), while simultaneously reducing transitions between the open state and a ~65-ps subconductance state. The mutation of Ser395 to alanine attenuated the PKA-mediated increase in GJIC and altered, but did not eliminate, the channel response to PKA. This study also showed that PKI, a specific PKA inhibitor abolished the increase induced by PKA activators. This phenomenon is consistent with a previous report from Calvin et al. [157], who showed that introducing large cortical cataracts with severe disturbances of lens electrolytes by H-89, an inhibitor of PKA. It is plausible that the primary cause of H-89-induced cataracts involves the inhibition of PKA phosphorylation.

## 6. Conclusions

**Figure 1:**
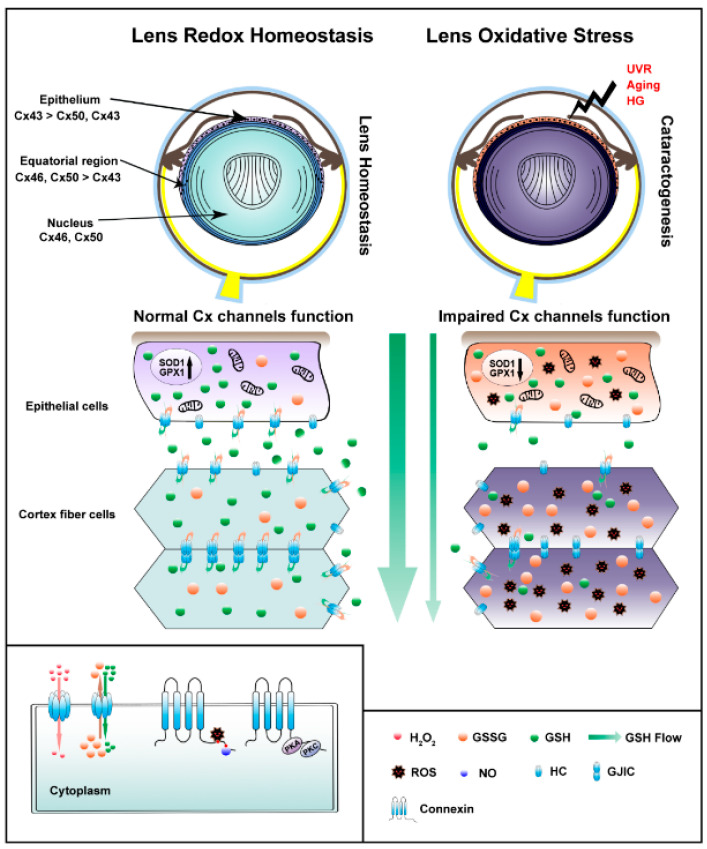
Illustration of connexin-forming gap junctions and hemichannels in the lens under normal and oxidative stress conditions. The distribution of connexin subtypes in various lens regions is shown (**upper panel**). At normal physiological conditions (**middle left panel**), the expression of antioxidant genes, such as superoxide dismutase 1 (SOD1) and glutathione peroxidase 1 (GPX1), is high, and glutathione (GSH) synthesized by lens epithelial cells is released possibly through connexin hemichannels. The extracellular GSH will be uptaken by cortical lens fiber cells through fiber connexin hemichannels and further delivered through gap junctions to inner lens fiber cells. The ratio of GSH and oxidized GSH (also known as glutathione disulfide, GSSG) is high in the normal lens. When the lens is continuously subjected to oxidative stress, due to (ultraviolet radiation) UVR, aging, or high glucose (HG) (**middle right panel**), expression of antioxidant gene expression is lower, and the biosynthesis of GSH is reduced in lens epithelial cells. This leads to reduced GSH release, and in the meantime, less GSH will be uptaken by cortical lens fiber cells. The reduced GSH level in lens cortical fiber cells will disrupt the balanced redox potential accompanied with elevated oxidant GSSG and reactive oxygen species (ROS), and lower GSH/GSSG ratio. Consequently, fewer reductants, but more oxidants will be transferred to the inner lens fibers through gap junctions. The compromised redox homeostasis will generate a vicious cycle, and resulted in elevated oxidative stress will lead to cataratogenesis. Connexins can be post-translationally phosphorylated at its C-terminus by protein kinases, such as protein kinase A (PKA) and protein kinase C PKC. Oxidative stress, such as H_2_O_2_ and NO, can alter connexin phosphorylation, leading to changes in connexin hemichannel function (**bottom left panel**).

As illustrated in Figure 1, growing evidence has unveiled the importance of GJIC and HCs in redox regulation and cataratogenesis in the lens. During aging, lens redox states gradually transition from being reductive to highly oxidative. This is associated with changes of expression and post-translation modifications of connexin molecules. The oxidative-induced alterations of connexin proteins decrease GJIC, leading to the accumulation of oxidants and consequently cataract formation, since antioxidant molecules cannot be delivered into the lens core. In contrast to GJIC, oxidative stress opens HCs, and this opening enables a cellular protective mechanism against oxidative stress-induced cell death. The oxidative stress on lens proteins, lipids, and DNA may result in an imbalanced redox environment and activation of signaling pathways that induce post-translational modifications of connexins and oxidation of connexin molecules. As a critical regulator of redox homeostasis, the responses of connexin channels to oxidative stress are complex, and the net effects likely depend upon multiple factors, such as phosphorylation, cellular redox state, and membrane potential. Studies regarding crosstalk between these factors will help to elucidate the relationship to oxidative stress, GJIC/HCs, and cataracts. In general, current evidence strongly supports the key roles of connexin channels in oxidative stress-related disorders in the lens, but the underlying molecular mechanisms between oxidative stress, connexin channels, and cataract formation require further investigation.
